# How to meet coping strategies and preferences of children during invasive medical procedures: perspectives of healthcare professionals

**DOI:** 10.1007/s00431-024-05802-1

**Published:** 2024-10-09

**Authors:** Elisabeth W. Segers, Agnes van den Hoogen, Lisette Schoonhoven, Elise M. van de Putte, Marjolijn Ketelaar

**Affiliations:** 1 Department of Children, Wilhelmina Children’s Hospital, University Medical Center Utrecht, PO Box 85090, 3508 AB Utrecht, The Netherlands; 2 Department of Neonatology, Wilhelmina Children’s Hospital, University Medical Centre Utrecht, PO Box 85090, 3508 AB Utrecht, The Netherlands; 3 Nursing Science, Julius Center for Health Sciences and Primary Care, University Medical Center Utrecht, Utrecht University, Utrecht, The Netherlands; 4 Faculty of Environmental and Life Sciences, School of Health Sciences, University of Southampton, Southampton, UK; 5 Center of Excellence for Rehabilitation Medicine, Brain Center Rudolf Magnus, University Medical Center Utrecht and De Hoogstraat Rehabilitation, Utrecht, The Netherlands

**Keywords:** Pain, Distress, Children, Healthcare professionals, Coping preferences

## Abstract

Children with negative procedural experiences have an increased risk of fear and distress, with psychological consequences for subsequent procedures and future healthcare behaviors. Gaining control and feeling trust are important aspects for children to decrease fear. To enable professionals providing personal care during medical procedures, a systematic, evidence-based approach that supports children in expressing their preferences is needed. This study will gain insight into the experiences and needs of professionals involved in small invasive medical procedures to meet the coping strategies and preferences of children undergoing these procedures. A qualitative design was used to gain insight into professionals’ perspectives. Data were collected through online focus groups with various professionals involved in medical procedures, such as anesthetists, laboratory staff, nurses, and pediatricians. Five interdisciplinary focus groups, with a total of 32 participants, were held. One overarching theme was revealed: “Balancing between different actors within the context of the hospital.” Professionals reported they had to deal with different actors during a medical procedure: the child, the parent, the colleague, and themselves. Each actor had its own interests. They were aware of the child and parents’ priority to gain control and feel trust. Nevertheless, they perceive organizational and personal aspects that hinder addressing these needs. *Conclusion*: To provide personalized care, professionals experience balancing between the needs and interests of diverse actors during medical procedures. The findings underscore the importance of a policy supporting HCPs in integrating patient-centered care into practice through practical tools and training initiatives such as scenario training.

**Table:** 

**What is Known:** • *Unresolved pain and stress arising from medical procedures can have significant short- and long-term impacts on children. Empowering children to gain control and fostering a sense of trust are crucial factors in reducing fear associated with medical procedures*. • *Children and parents expect to receive child-tailored care from professionals including respect for their own, unique needs, and boundaries. Professionals should build trustful relationships and provide appropriately tailored autonomy around medical procedures*.
**What is New:** • *Healthcare professionals vary in their awareness of children’s needs during a medical procedure. Beside this, the organizational dynamics of the hospital, along with the presence of actors such as the child, parent, colleague, and oneself, collectively influence the conduct of medical procedures*. • *Providing person-centered care during medical procedures can present challenges. The results highlight the significance of a policy to assist healthcare professionals in incorporating patient-centered care into their practice through practical tools and a culture of self-reflections regarding patient-centered values*.

**What is Known:**

• *Unresolved pain and stress arising from medical procedures can have significant short- and long-term impacts on children. Empowering children to gain control and fostering a sense of trust are crucial factors in reducing fear associated with medical procedures*.

• *Children and parents expect to receive child-tailored care from professionals including respect for their own, unique needs, and boundaries. Professionals should build trustful relationships and provide appropriately tailored autonomy around medical procedures*.

**What is New:**

• *Healthcare professionals vary in their awareness of children’s needs during a medical procedure. Beside this, the organizational dynamics of the hospital, along with the presence of actors such as the child, parent, colleague, and oneself, collectively influence the conduct of medical procedures*.

• *Providing person-centered care during medical procedures can present challenges. The results highlight the significance of a policy to assist healthcare professionals in incorporating patient-centered care into their practice through practical tools and a culture of self-reflections regarding patient-centered values*.

## Introduction

Many children experience pain and distress prior to and during medical procedures, possibly with major short- and long-term consequences. For example, needle-related procedures, as well as minor procedures such as inserting or removing urinary catheters or removing plasters, can be experienced as painful and distressing in children [1, 2]. This procedural distress can have a major impact on the hospital experience of sick children and their parents, resulting in anticipatory anxiety and distrust in healthcare professionals (HCPs) and can even develop into a post-traumatic stress syndrome. Subsequently, it might have an impact on an organizational level, such as a delay in performing necessary procedures and pressure on HCPs to use restraint [3, 4].

An evidence-based management plan to minimize procedural pain and distress is therefore crucial in healthcare organizations. Such a plan not only consists of pharmacological interventions which reduce pain or lower consciousness but also includes psychological and non-pharmacological approaches [5, 6]. These interventions must be appropriate to the child’s developmental level and result in distracting the child’s focus of attention from the procedure [5, 7–9]. The central theme is the need to develop a trustful relationship between the child, parents, and HCPs and to experience autonomy during a medical procedure [10, 11]. Therefore, in order to provide adequate pain and distress management, HCPs must be aware of the child’s personal needs and involve children (and their parents) when making child-tailored choices and decisions in procedural care.

HCPs have an important role in guiding children and parents during procedures [12]. Although HCPs in general are aware of this importance, research shows that they often underestimate procedural pain and distress in children. Moreover, HCPs performing a painful procedure tend to disconnect themselves from the pain the procedure may cause [13, 14]. As a result, they may not consistently recognize the significance of individualized, patient-focused care to mitigate pain and alleviate fear during procedures. Therefore, it is a prerequisite for HCPs to be aware of their own needs and reflect on their behavior in order to implement appropriate interventions that support children and parents in a tailored manner. The development of evidence-based approaches that support children and parents would enable HCPs to provide personalized care during medical procedures, aligning with the preferences of children and parents.

This study aims to gain insight in the experiences and needs of HCPs in supporting children undergoing minor invasive medical procedures. It provides valuable insights to enhance their competence in meeting the coping strategies and preferences of the children in order to minimize pain and anxiety during these procedures.

## Methods

### Design

A qualitative study was conducted to obtain insight into the perspectives of HCPs using semi-structured online focus group interviews. These interprofessional focus groups were organized to discuss experiences and needs and to stimulate an interdisciplinary debate about HCPs demands to support children and parents and to meet children’s coping strategies during a medical procedure.

### Study setting

The study was conducted at a tertiary care pediatric university hospital in the Netherlands. HCPs were recruited between March and June 2021. The Medical Research Ethics Committee of the UMC Utrecht confirmed that the Dutch Medical Research Involving Human Subjects Act (WMO) did not apply to this study.

### Sample, recruitment, and data collection

Selected participants were HCPs, employed in inpatient departments of the pediatric university hospital and involved in minor invasive medical procedures in a hospital: clinicians (anesthetists, pediatricians), nurses, child life specialists, radiology, and laboratory staff. There was a strive for variation in the number of years of work experience among the included HCPs.

To recruit participants, an invitation was published in newsletters of various hospital wards. Participants included HCPs who reached out to the research team, as well as HCPs who were specifically asked to participate by members of the research team. The final selection of HCPs was criterion based and purposive, to ensure the representation of the various medical professions in each focus group in order to gather a wide range of perspectives. Included HCPs received an information letter on the study purpose, procedure, and confidentiality. Written informed consent of each participant was given before the start of the focus groups.

Next to the information letter, a vignette was sent to each participant in preparation of the group interview. This vignette contained a summary of lived experiences from various children and parents of children undergoing minor invasive medical procedures such as intravenous blood sample, intravenous infusion, gastro nasal tube insertion, or subcutaneous injections. The citations are findings of an earlier study on children’s and parents’ experiences, needs, and wishes regarding coping strategies around minor invasive medical procedures (Appendix 1) [10].

A semi-structured topic guide was developed by the research team, which was informed by the vignette and clinical experiences (Appendix 2). Guiding questions were designed to prompt views on the following:

-Perceptions and experiences of the HCPs regarding the needs of children and parents around medical procedures.
-Needs of the HCPs to support children and to meet their wishes, along with the barriers and facilitators they perceived in this process.

In the fifth focus group, no additional information related to the themes was identified and enrollment stopped [15, 16]. Each focus group consisted of four to eight members representing various HCPs involved in medical procedures.

The interviews were conducted by a neutral moderator of the Department Social Care in the hospital, with experience in leading focus groups and not involved in the design or execution of the study, nor a colleague or supervisor of the participating healthcare professionals, and a researcher (EWS) acting as an assessor, listening actively, posing following up questions if required and summarizing. During the interviews, field notes were made by the assessor (EWS).

### Data analysis

The focus group interviews were audio-recorded, transcribed verbatim, anonymized, and analyzed with an inductive thematic analysis approach [17]. The interviews were read and reread; initial coding and analysis were done by EWS, AH, and MK separately, followed by a discussion on the meaning of the quotes. Themes and subthemes then were developed based on codes, analytical memos, and feedback from research team discussions. Coding, memos, and analyses were documented digitally.

Consistency of the code scheme was checked by LS and EP after which discussion ensued regarding the themes, and some changes were proposed and adapted. Interpretations were iteratively reviewed and critically discussed until a consensus was reached within the research team. The report was written according to the consolidated criteria for reporting qualitative research (COREQ) guidelines [18].

## Results

Five focus groups, with a total of 32 participants, were held between April and June 2021. In each group, different disciplines participated. In total, 2 child life specialists, 3 radiology staff members, 4 laboratory staff members, 4 anesthetists, 6 pediatricians/in residence, and 13 pediatric nurses participated in the study (see Table 1). Work experience of the HCPs varied from 1 to 32 years. The focus groups, which were online due to the COVID-19 pandemic, lasted 60–75 min and were in Dutch.

**Table 1:** Characteristics of healthcare professionals

Characteristics of the healthcare professionals (*N* = 33)	
**Profession**	**N**
Anesthetists	2
Anesthesiology staff	2
Child life specialist	2
Laboratory staff	4
Pediatrician (in resident)	6
Pediatric nurse	13
Radiology staff	2
**Years of experience**	
< 5	20
5–15	7
> 15	6

Each focus group started with a prompt to elicit reactions on the vignette (see Appendix 1). HCPs acknowledged the needs of children and parents. However, throughout the interview, HCPs discussed their insights regarding various experiences and challenges to fulfill these needs. Their own needs emerged less frequently during the interviews, despite being explicitly queried.

### Overarching theme and subthemes

During the analysis of the interviews, one overarching theme emerged, namely: “Balancing between the different actors within the context of the hospital.” The different actors are the child, the parent, the colleague, and the individual HCP.

**Fig. 1:**
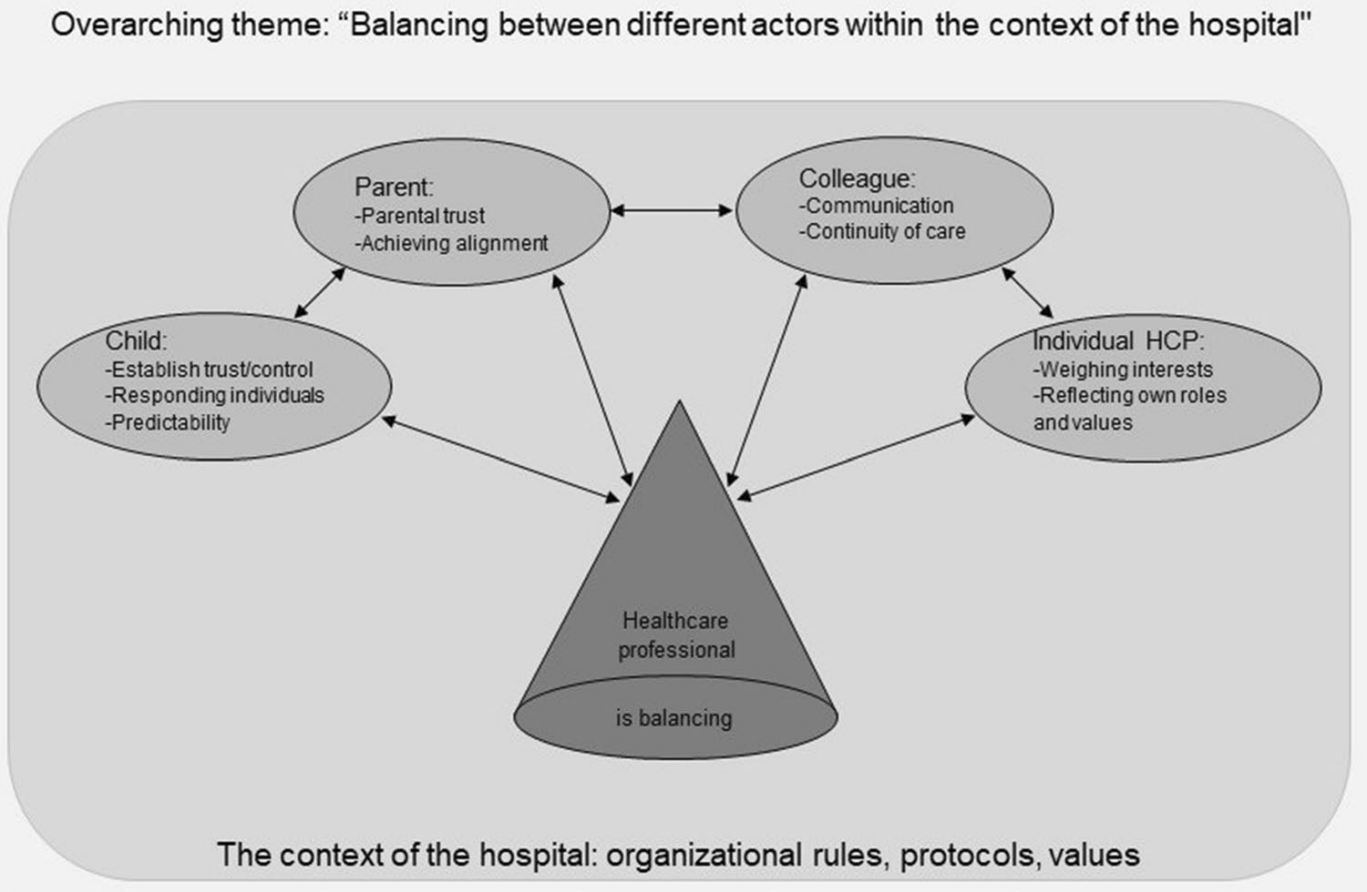
Overview of the overarching theme, the actors, and the subthemes

To organize the findings, the subthemes were categorized by the different actors, as the interviews revealed that the professionals’ experiences were interconnected with these various actors. Under each actor, the associated subthemes were described. See Fig. 1 for an overview of the overarching theme, the actors, and the subthemes.

### Balancing between the different actors

The HCPs perceived the context of the hospital and the different actors involved in a medical procedure as co-determining the conduct of the medical procedure. They recognize the needs of the child; nevertheless, the context of the hospital and the other actors (including themselves) have their own rules, needs, and demands that exert pressure on them and influence the course of the procedure.

HCPs observed that if the needs of an actor are not met or if an actor is not able to cooperate effectively, the medical procedure does not proceed as intended. The impact of this can vary for children and parents. Nevertheless, the interviewed HCPs felt responsible for the course of the procedure and consequently recognized the complex task of maintaining the balance between diverse needs and the interests of the multiple actors to effectively manage a medical procedure.

### The context of the hospital

The hospital organization is the setting in which medical procedures take place. HCPs operate within a context of rules, protocols, and values established by the organization. However, by adhering to this, the activities of HCPs also expose patients and parents to the organizational context.

HCPs sometimes experienced conflicts between efficiency and patient-centered care because they could not always take the time for children who were very anxious and could not always accommodate the wishes of the child and parents.

Anesthetist 1: So yes, that went well because we took a lot of time, you know, we were able to avoid the struggle. But that is very nice, but it has enormous consequences for the whole operation rooms schedule, which is always tight, and yes, delays mean overtime and so that is all difficult.

PedNurse7: Honestly, we don’t really have the time for that. I once spent time trying to alleviate a child’s fear, but it took me an hour and a half during my evening shift.

### The child

Three subthemes are identified pertaining to the child as an actor: establishing trust, responding to individual differences, and providing predictability. These themes are considered key needs of children to optimally support them during a medical procedure.

At first, the HCPs recognized the crucial importance of establishing trust in children undergoing medical procedures. They incorporated this consideration through various strategies, acknowledging that building trust requires careful attention to the child’s desires and requirements, especially in terms of coping mechanisms. Moreover, emphasis was placed on HCPs adhering to predetermined agreements and maintaining transparency when deviations from the agreed-upon course became necessary. The cultivation of trust was facilitated by engaging in discussions about everyday subjects that captured the child’s interest, serving the dual purpose of diverting attention from the medical procedure and fostering a relational connection between the HCPs and the child, thereby nurturing trust.

Anesthetist 2: Because, of course, we do a lot of things that are exciting and scary for many children, and it is very nice to be able to engage with something you know the child likes or has hobbies… Yes, the comfortable chat that can make it more personal, we don’t really know that about every child, and sometimes it goes very easily, and sometimes you think: yes, that was not exactly what I should have said to this child.

PedNurse 8: Yes, and keeping to agreements, keeping to agreements.

Pediatrician 3: Yes, and not making false promises.

Secondly, HCPs observed that each child possessed unique needs and preferences, which could vary from one instance to another. Additionally, a child’s coping strategy often changed, even during the procedure, as a particular approach might no longer be effective. This implies that HCPs could not rely on fixed standards but had to remain attentive to evolving circumstances. This demands keen observation and the ability to adapt swiftly, which is not easy in hospital settings where interactions between HCPs and patients are often brief and infrequent.

Labstaff 3: Because suddenly you have them in your treatment room, and at that moment, you have to quickly figure out what this child wants. How can I reassure them, and that is sometimes quite difficult.

PedNurse 8: But you see that care is personalized, right? What is pleasant for one person is not for another. And it is just difficult to figure that out.

HCPs also recognized the advantageous impact of affording children a measured degree of control. This involved actively soliciting the child’s preferences and allowing them to articulate their accustomed approaches. Simultaneously, HCPs acknowledged the potential deleterious effects of too much control, which may induce anxiety in children confronted with too many options. They argue that the provision of control should be matched to the child’s developmental capacity, considering their age, developmental stage, and previous experiences. The delicate task of striking an appropriate balance in giving control during medical procedures is perceived by HCPs as intricate, given that both excessive and insufficient control can cause anxiety in children.

Anesthetist 2: Well, we often give them a choice. But then it’s about where to place the saturation meter. So it’s like: on which finger shall I put it, or on which toe? … By doing this, you hope that they have a bit of control, that they get to choose something.

Anesthetist 3: Yes, because I think that counting down to give them some control, placing full control with the child, doesn’t always yield the best result. I want to give control with the child’s participation, starting from that position, but not to the extent that we lose or give up control. Indeed, with those consequences for safety.

### The parent

HCPs indicated two subthemes associated with the parental role in the context of a medical procedure: the paramount importance of cultivating trust with parents and the necessity of achieving alignment with them to foster collaborative engagement. HCPs perceived parents to be crucial partners in effectively supporting a child during a medical procedure. Parents share a close bond with their child and possess an unparalleled understanding of their child. Nonetheless, the prospect of a medical procedure for their child can be an anxiety-inducing experience for parents as well. HCPs noted that this can negatively impact the child. They recognized that parents have specific needs that should be taken into consideration.

During the focus group sessions, emphasis was placed on the significance of garnering parental trust not only to establish confidence with parents but also to extend that trust to the child. HCPs, akin to their interaction strategies with children, employ “small talk” as a means to engage with parents and cultivate trust. Moreover, HCPs recognize that demonstrating expertise plays a pivotal role in trust-building with parents. This expertise is manifest in interactions with the child, discerning appropriate communication and fulfilling commitments. Furthermore, accurate and flawless execution of medical procedures, coupled with adequate information, contributes to parents’ trust in the HCP. HCPs noted that when parents have confidence in the procedural setting, it helps the child more easily develop trust in their interactions with the HCPs.

PedNurse 2: By carefully observing what I learn from children and parents, what works for them, it also works for me. Creating trust with parents is indeed important.

PedNurse 7: I think it’s also important to gain the trust of parents, so the child feels you are trustworthy. Then, winning the child’s trust by having a chat and relaxing a bit.

Most of the HCPs underscored the significance of aligning with parents to establish a collaborative approach during procedures. When there is disagreement with the parent, for instance, if the parent not fully endorses the procedure or prefers an alternative approach or mode of guidance, it often has adverse effects on the child’s trust, possibly resulting in a more arduous procedural experience. Negotiating this delicate situation can pose challenges for HCPs who may find it difficult to express dissent while simultaneously preserving the trust and rapport with parents. HCPs stressed the need to take a leadership role during procedures, but at the same time working together and staying aligned with parents is crucial.

PedNurse 2: Yes, the best thing is if you can also talk with parents about what role they can take. What will work, how do they know their child, and what will help their child the most?

Anesthetist 1: Because you also want to get a reaction from the parents, like, okay, this is going in the right direction, it’s going to work, and this is how we’ll do it. So, we indeed need to collaborate on this.

### The colleague

Two subthemes were identified under another actor “the colleague”: communication and continuity of care. Apart from the child and the parent, HCPs recognized their collaborating colleague(s) as a specific actor during a medical procedure. While working with colleagues, HCPs encountered two distinct challenges.

Firstly, HCPs recognized the importance of exchanging essential information before the medical procedure when guiding a child. This information typically includes personal details such as coping rituals, specific preferences, interests, fears, and agreements made with the child and parents regarding a procedure. This information can assist HCPs in building a trusting relationship with a child, which is crucial for the smooth progress of a procedure. In a hospital setting, where various disciplines are at work and schedules may be irregular, HCPs noted that this information is not always effectively communicated. During the focus group interviews, discussions arose about finding a well-defined location for this information within the information systems used in a hospital, such as an electronic patient record.

PedNurse 3: So that you know what has been implemented in the department and can continue with it. Sometimes I think we are all isolated, and we need to collaborate more to properly support a child, yes, yes.

Anesthetist 2: And it is very nice to be able to engage with something you know the child likes or their hobbies, or if they have been able to think about it at home. I thought: maybe you could make a sort of form to fill out with things they like. So they have already thought about it together, parent and child.

Additionally, HCPs observed that even when information and agreements about a patient are effectively transferred, it does not necessarily mean that the resulting policies are consistently continued by colleagues. HCPs noted that this is influenced by various factors: the HCP taking over the care may have different priorities, such as time constraints due to a tight schedule, and may not always perceive the urgency of the information. Furthermore, HCPs had varying perspectives on certain commitments made to children regarding the execution of procedures. Failure to communicate about these differences led children and parents to realize that HCPs do not always adhere to agreements made by others, which can impact the trust relationship.

PedNurse 7: Sometimes I hear from nurses who have everything together and then they go to the OR, for example, and then they do things completely differently. Even though you have asked them to do so and so, and then they say no we don’t do that and I think oh boy.

### The healthcare professional

The interviews revealed that the HCP, as an individual, is also a participant in the interplay among various stakeholders and crucial for the course of a medical procedure. What the participating HCPs mentioned about their own role can be summarized into two subthemes: weighing interests and reflecting on their own role. Additionally, HCPs expressed certain needs regarding guiding children and parents.

In order to provide quality care during medical procedures, HCP navigate between the diverse interests of the child, the parent, the organization, and their own. As previously described, HCPs generally recognized the necessity of taking the child’s interests seriously and building a trust relationship, viewing it as an investment that pays off later. However, HCPs often feel the pressure of their own agenda, influenced by organizational needs and the needs of the other actors as described earlier. The choice made by the HCP in that moment, and which side of the balance tips, appears to be determined by various factors: the skills and values of the HCP, the time pressure they experience, the bond with the child and the parent, and the collaboration with other HCPs at that moment.

Labstaff 1: It’s otherwise recognizable, yes, so a lot of things recognizable that don’t always deserve a beauty prize and the other side I also say can’t always do it either with the time pressure it’s, it’s a little bit. Yes. A bit of give and take. If it already takes a little longer, then you think of well come, shall I just do it now?

PedNurse 7: Well for example asking parents to be very patient and to count to 10 before a child does something, but you are someone who is very eager to get on with it and sees that it only gets worse and that it doesn’t progress like that….. then you have to get further away from yourself so that it doesn’t feel like your own anymore so to speak.

During the interviews, many HCPs engaged in self-reflection regarding their roles during medical procedures. Throughout all focus group interviews, it emerged that at times, they make the choice to restrain a child against their will during a procedure, leading to a subsequent sense of failure. When tension rises and the HCP is uncertain about how to proceed, there is a tendency to act quickly. On the other hand, some questioned whether accommodating a child’s needs is always the best choice during a procedure, even for the child’s benefit. HCPs pondered during the interviews whether they truly perceive each child as unique or if they tend to standardize too quickly and approach children too routinely.

Due to the need to quickly weigh interests during complex situations in a medical procedure, HCPs expressed a desire for more skills in building trust with the child and the parent. They sought guidance on handling difficult social interactions during a procedure. HCPs advocated for scenario training and opportunities for reflection after a procedure, recognizing that these tools could enhance their ability to navigate challenging situations and make informed decisions.

PedNurse 1: Because I do have the idea that sometimes we are going to do things through a schedule, so that that I do approach children less individually than if I wanted to do it.

PedNurse 2: So it’s always a bit of searching for awareness: how do I get to the togetherness, what do they have for experience, what do they need and what can I offer to make it go well?

HCPs expressed specific needs to guide children and parents during a medical procedure. It was emphasized that it would be helpful to easily discern the preferences and coping rituals in order to respond more effectively in a manner tailored to the child. Additionally, HCPs questioned how to address children who experience intense fear and resistance. Should HCPs align with the child’s preferences (to avoid conducting the procedure), or are there alternative approaches to proceed with the medical procedure without resorting to restraining the child?

PedNurse 10: Then it is very difficult to go along with them because they are afraid of it. So then yes, to what extent should you take their wishes into account, that is quite difficult to see a happy medium in that.

Additionally, HCPs expressed a desire for enhanced skills themselves to better support parents in challenging situations, such as feeling pressured by parental expectations. Furthermore, they believe that as HCPs, they should be more conscious of the specific role of the parent during a procedure, as described above.

PedNurse 4: What we do, for example on the ward, we do scenario training, but those are always acute situations, medical interventions, but never difficult situations with parents, yes, that’s on, we don’t think that’s important enough. Whereas that definitely does occur very often as well and we assume that everyone would be able to do that (…). It would be nice to have tools for that though, or an education.

## Discussion

This study highlights that HCPs perceive themselves as balancing between different actors within the hospital context during medical procedures involving preferences of children. The organizational dynamics of the hospital, coupled with the presence of actors including the child, parent, colleague, and the HCP, co-determine the conduct of medical procedures. How the balance can tip is influenced by a myriad of factors, including the skills and beliefs of the HCP, organizational values and schedules that may impose time constraints, the bond with the child, the role of parents, and the collaboration with colleagues within the organization. These factors can lead to failure in addressing the wishes and needs of the child.

This article focuses on the needs and challenges of professionals in meeting the preferences of children during medical procedures, which is part of shared decision-making (SDM). SDM promotes collaboration between patients, family members, and HCPs in the context of patient-centered healthcare [19].

There is limited knowledge about the application of patient-centered care and shared decision-making regarding medical procedures in children as well as how to support HCPs in integrating patient-centered care in this practice. Research does exist, however, on the challenges that can arise between the concepts of family-centered care and child-centered care, especially when professionals focus primarily on collaborating with parents, which can lead to the needs and rights of the child being overlooked [20, 21]. Bray et al. show this in a study, investigating how healthcare professionals handle the restraint of children during clinical procedures [22]. Similar to the overarching theme in our study, Bray et al. emphasize that healthcare providers perceive themselves as balancing between different stakeholders, including parents, during a medical procedure, resulting in a lack of consideration for the child’s interests and wishes and the child being restrained to perform the medical procedure. HCPs often expressed feeling uncomfortable when this occurs. Bray et al. propose a practical solution to promote a more balanced approach in clinical practice. They suggest the implementation of a “clinical pause” during a medical procedure, which would give healthcare professionals the necessary time to consider children’s expressed wishes and explore alternative approaches to restraint.

Some more specific subthemes, mentioned by the HCPs in our study, such as the need for a trustful relationship between the child/parent and the HCP, the need for training in communication skills, and insufficient time to address patient preferences, are also described in studies on pediatric shared decision-making (SDM) in a hospital context [23, 24]. Although these studies typically focus on decisions regarding the treatment of the child’s illness during conversations between doctors and parents, rather than on experiences related to medical procedures amidst the hectic environment of a hospital ward, they similarly identify challenges mentioned by HCPs in our study. Practical strategies mentioned include training in communication skills, such as listening and actively inquiring about preferences, as well as developing decision aids for parents and children [24].

Additionally, these studies highlight a significant barrier that is not explicitly mentioned in our study but appears to be present: the lack of awareness of patient-centeredness and SDM among HCPs. In our study, this was particularly highlighted in the actor “the HCP,” under the subtheme “weighing interests,” which revealed that HCPs weigh various interests and do not always prioritize patient-centered care. The question can be raised as to why HCPs struggle to put this concept into practice. Anderson and Funnell stated that patient-centered care is a relatively new concept in healthcare [25]. New concepts are based on new values, and it takes time to internalize these values. Often, HCPs are not aware of their own values regarding personalized care [26–28]. This means that, in addition to practical skills training, it is also necessary to encourage and guide HCPs in reflecting on their values and motivations. By integrating such interventions as reflection on one’s own actions and preparation before and after a medical procedure into clinical practice, healthcare organizations can cultivate environments that prioritize patient-centered care and improve the overall quality of care for pediatric patients and their families [26].

The strengths of our study include a comprehensive qualitative approach according to the COREQ guidelines and the use of investigator triangulation to enhance credibility and trustworthiness [18].

However, we acknowledge some study limitations. While the moderator was careful and no single participant dominated any group, it is possible that perceptions of influence may have encouraged some to participate more than others. Finally, regarding the population of professionals recruited to participate in the focus groups, certain disciplines such as nurses were better represented than others such as child life specialists, radiology, and laboratory staff. This could have potentially influenced the outcomes, although they did not express markedly divergent viewpoints during the interviews.

### Clinical implications and further research

The outcomes of this study underscore the need for support for HCPs to deliver truly patient-centered care. In the focus groups, HCPs highlighted practical considerations such as the implementation of tools that allow for the incorporation of patient preferences into electronic medical records, clinical pauses before and during a medical procedure, and scenario training sessions focused on managing challenging situations, such as dealing with highly anxious children. The literature further indicates that, in addition to skill-building training, reflecting on one’s own values regarding patient-centeredness is crucial for further integrating patient-centeredness into clinical practice. New research into the needs of professionals and the impact of practical skill-building training, along with reflection on the values underlying patient-centered care, is needed.

In conclusion, the findings of this study underscore the complex interplay between various actors within the hospital context when striving to achieve patient-centered care, meeting the preferences and coping strategies of children during medical procedures. HCPs often find themselves balancing between the needs and preferences of children and parents, collaboration with colleagues, demands of the organization, and their own professional values. The findings underscore the importance of supporting HCPs in integrating patient-centered care into practice through practical tools and training initiatives. Furthermore, fostering a culture of self-reflection regarding patient-centered values is crucial for further advancement. By addressing these challenges and promoting a patient-centered approach, healthcare organizations can enhance the quality of care delivery and ultimately improve patient outcomes.

## Data Availability

No datasets were generated or analysed during the current study.

## Appendix 1. Vignette preparation focus group interview

### Preferences and rituals that children develop during unpleasant medical procedures

This interview is part of a series of studies aimed at creating a new tool to make it easier for children to choose and express what they like when undergoing an unpleasant procedure.

Children and parents, as revealed in a previously conducted qualitative research, highlight two themes as crucial during (regularly occurring) unpleasant procedures:

*To gain control and to feel trust*

**Statements from children and parents on these themes:**

“It’s just nice when it’s done, and I can say… yeah, that they count down for a moment. Yeah, I don’t know, I always do that, I just find it comforting” (Boy, 14 years old, regularly intravenous infusions).

“Just tell what needs to be told and don’t make it bigger than it is” (Boy, 16 years old, intravenous infusions).

“Don’t start right away with, ‘we’re going to prick you, and this and that is going to happen.’ But just start by having a nice chat with someone” (Mother of a 15 years old boy, subcutaneous injections).

“They all did it in their own way. While I think, ‘hey, it’s fine that you have your own way, but we need to look at X and listen to X” (Mother of a 11 years old girl, intravenous infusions).

“Well, everyone knows here how I want it, that gives a sense of calm, actually” (Boy, 17 years, gastro nasal tube insertions and intravenous infusions).

“So, he could choose between the ring finger and the middle finger or something. But then he didn’t choose immediately. So, they said, ‘well, then we’ll just pick one, done.’ You know? Well, that just doesn’t work” (Mother of an 8 years old boy, intravenous blood sample).

“Yes, I find injections scary, and when I’m poked badly too, then it’s even more unpleasant” (Girl, 10 years old, intravenous infusions).

“So, I just think it’s best if a nurse knows in advance how the person wants it. For example, I don’t need a splint, there’s no need to count with me” (Girl, 17 years old, intravenous infusions).

“And if you do that very often, you’ll eventually figure out what works, yes” (Girl, 14 years, intravenous infusions).

“Yes, and you can already tell that after the last injection… that it sticks, so to speak. While he found the first two very unpleasant, too, but you can tell that the most unpleasant things linger” (Mother of a 6 years old boy, intravenous blood sample, infusions, nasogastric tube insertions).

**Consider the following questions in advance:**

- What do you think of the needs expressed by children and parents in the above quotes?
- How can you contribute to these needs?
- What do you need to align with or take into account the preferences/rituals of a child?
- What do you need to ensure that children and parents have confidence in you?

## Appendix 2. Topic list focus group interviews with hcps

With the interviews we are about to conduct, we aim to clarify what you as healthcare providers need to consider regarding the preferences of children and parents in your care for pain and anxiety reduction.

- What is your opinion on the needs expressed by children and parents in the above quotes?
- How can you contribute to meeting those needs?
- What do you need to ensure that children and parents trust you?
- How can you give the child a sense of control while maintaining control yourself?
- Do you ever teach a child a way to make a procedure more pleasant, thus developing their own preferences? What do you need to be able to do that (skills, repertoire)?
- What do you need to connect with or consider the preferences/rituals of a child?
- In what way can we transmit, preserve/store, and find children’s wishes/rituals?
- Alternatively, what do you need to find out what the wishes of the child and parents are? How do you currently do this?

## Abbreviations

HCP: Healthcare professionals
UMC: University Medical Centre
WMO: Wet Medisch-Wetenschappelijk Onderzoek (Dutch Medical Research Involving Human Subjects Act)
